# Supplementary material for: Histidine-Rich Defensins from the Solanaceae and Brasicaceae Are Antifungal and Metal Binding Proteins
Source: J Fungi (Basel). 2020 Aug 24;6(3):145. doi: 10.3390/jof6030145 (PMC7557933; doi:10.3390/jof6030145)

Table S1: Primers used for cloning CDS into pPink

PRIMER NAME	SEQUENCE 5' TO 3'
**ATD90_FWD**	TCTCTCGAGAAAAGAGCTCATTGTGATCACTTCTTGGGAGA
**ATD90_REV**	GAGCTCGGTACCTTAATGGTAATCTCCATAGCAGTGACA
**ATD212_FWD**	TCTCTCGAGAAAAGAGCTAACTGTGATACCTATCTTGGAGAGG
**ATD212_REV**	GAGCTCGGTACCTTATCTACCGTAGCAGTGACA
**CRD26_FWD**	TCTCTCGAGAAAAGAGCTAATGATTGTGATAGATTTCTTGGAGA
**CRD26_REV**	GAGCTCGGTACCTTAATGACCATAGCAATGACAGTGC
**SLD26_FWD**	TCTCTCGAGAAAAGAGCTAAGCATTGTGGAAAGCACTCT
**SLD26_REV**	GAGCTCGGTACCTTAGCAGTGGTAATAGCAGTAACA

Table S2. Statistical analysis of HRD structures[a]

	AtD90	SlD26
Experimental restraints		
total no. distance restraints	552	419
intraresidue	165	115
sequential	187	143
medium range, *i-j*<5	67	43
long range, *i-j*≥5	133	118
hydrogen bond restraints	16	24
dihedral angle restraints		
phi	38	33
psi	33	21
chi1	12	13
Deviations from idealized geometry		
bond lengths (Å)	0.011 ± 0.000	0.011 ± 0.00
bond angles (deg)	0.968 ± 0.031	1.001 ± 0.041
impropers (deg)	1.23 ± 0.08	1.35 ± 0.15
NOE (Å)	0.009 ± 0.002	0.010 ± 0.001
cDih (deg)	0.085 ± 0.069	0.037 ± 0.049
Mean energies (kcal/mol)		
overall	-1832 ± 55	-1514 ± 52
bonds	21.2 ± 1.8	19.8 ± 1.4
angles	52.2 ± 3.7	49.9 ± 4.5
improper	23.0 ± 2.6	24.3 ± 4.6
van Der Waals	-271.7 ± 8.5	-226.4 ± 9.2
NOE	0.05 ± 0.02	0.05 ± 0.01
cDih	0.12 ± 0.14	0.03 ± 0.05
electrostatic	-1898 ± 55	-1595 ± 58
Violations		
NOE violations exceeding 0.2 Å	0	0
Dihedral violations exceeding 2.0 Å	0	0
Rms deviation from mean structure, Å		
backbone atoms	1.37 ± 0.35	1.49 ± 0.31
all heavy atoms	2.20 ± 0.35	2.55 ± 0.38
Stereochemical quality[b]		
Residues in most favoured Ramachandran region, %	96.5 ± 2.1	94.1 ± 3.8
Ramachandran outliers, %	0.1 ± 0.4	0.1 ± 0.5
Unfavourable sidechain rotamers, %	0.9 ± 1.1	0.4 ± 0.9
Clashscore, all atoms	9.1 ± 3.6	5.9 ± 1.8
Overall MolProbity score	1.7 ± 0.2	1.7 ± 0.2

[a]All statistics are given as mean ± SD.

[b]According to MolProbity (Chen et al 2010).

Chen, V.B., et al., *Molprobity: All-Atom Structure Validation for Macromolecular Crystallography.* Acta Crystallographica. Section D, Biological Crystallography, 2010. **66**(Pt 1): p. 12-21.

Figure S1: Sequence alignment of selected *Nicotiana* and *Arabidopsis* defensins

```
A  NbD2      KYCWKKNHKWHGPCHYSYKCNHHCKHYFGAEYGVCKKYQWGHKHHHWAKYACYCYSPC His 10
   NbD1      KYCWKKSHKWHGPCHYSYKCSHHCKHYFGAEYGICKKYQWGHKHHHWAKYACYCYSPC His 10
   NbD3      KYCWKKSHKWHGPCHYSYKCSHHCKQYFGAEYGICKKYQWGHKHHHWAKYACYCYSPC His  9
             ******.*************.****:*******:************************

B  AtD90      HCDHFLGEAPV-YPCKEKACKSVCKEHYHHACKGECEYHGREV-------HCHC His  8
   AtD212     NCDTYLGEVTVYYPCRERDCEAQCYEHYPHSCKGECEHHDHVVHHDNEEEHCHC His  9
              :** :***. * ***:*: *:: * *** *:******:*.: *       ****
```

(A) Sequence alignment of the three HRDs initially identified from *Nicotiana benthamiana*.

(B) HRDs subsequently identified from *Arabidopsis thaliana*. Identical residues are indicated by an asterisk, similar residues are indicated by a full stop under the relevant column. Sequence alignments were performed using Clustal Omega (https://www.ebi.ac.uk/Tools/msa/clustalo/).

Figure S2: Graphical alignment overview to highlight cysteine and histidine residue distributions for Solanaceae and Brassicaceae histidine-rich defensins (HRDs).

Cysteines are indicated in yellow, histidines in blue, all other residues shown in grey. HRDs in the Solanaceae cluster (A) and the Brassicaceae cluster(B), and those that sit outside of those clusters (C) have differing cysteine residue organisation and distributions of histidine residues.

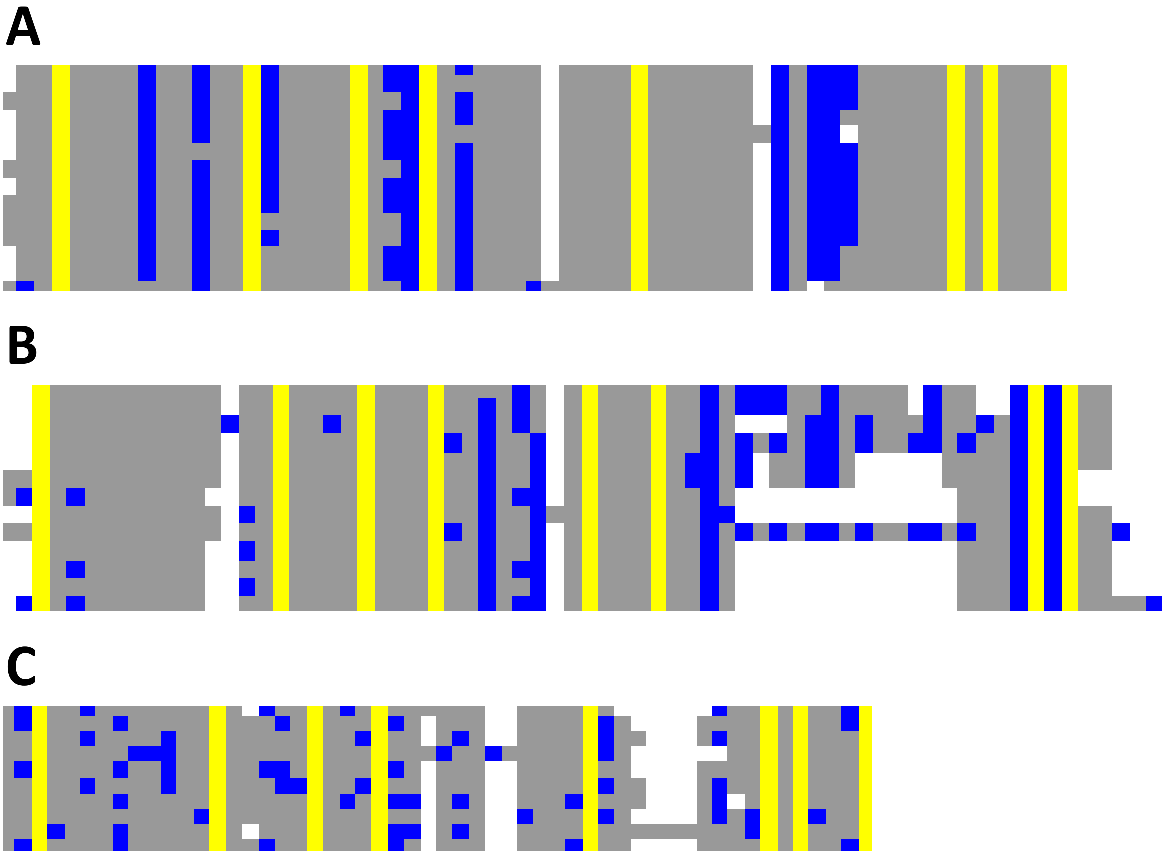

Figure S3: Tissue-specific gene expression of selected HRDs.

Transcriptome data was colelcted from public databases for three selected histidine rich defensins. Relative expression levels are drawn as coloured tissues, from white (no detected expression) to red (maximum expression), representing percentage expression for that gene. NbD2 tissues are (clockwise from top right position) flower, capsule, leaf, seedling, roots, main stem and apex. For AtD90 and AtD212, tissues are labelled on the diagram, shoot apical meristem is abbreviated as SAM.

Gene expression tissue map graphics were downloaded from the Nicotiana benthamiana Genome and Transcriptome sequencing consortium browser (NbD2 gene, aka transcript ‘Nbv6.1trP18806’)) and from the Tair *Arabidopsis thaliana* genome database (AtD90 ‘AT3G5727’ and AtD212 ‘AT3G05730’ genes). The Arabidopsis gene expression data was generated by Klepikova et al (2016), while the version 5 *Nicotiana benthamiana* transcriptome data was generated by Nakasugi et al (2014).

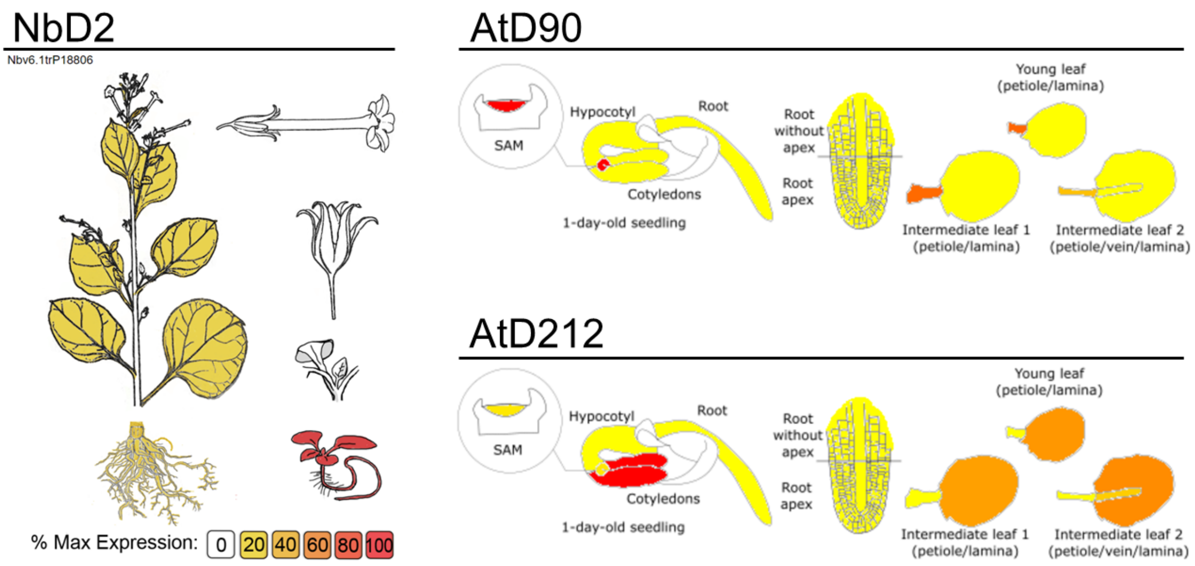

The Arabidopsis Information Resource (TAIR).

https://www.arabidopsis.org/servlets/TairObject?id=35604&type=locus

Klepikova et al (2016) Plant J. 88 1058-1070

Nicotiana benthamiana Genome and Transcriptome sequencing consortium

https://benthgenome.qut.edu.au/

Nakasugi K, Crowhurst R, Bally J, Waterhouse P (2014) Combining Transcriptome Assemblies from Multiple *De Novo* Assemblers in the Allo-Tetraploid Plant *Nicotiana benthamiana*. PLoS ONE 9(3): e91776. https://doi.org/10.1371/journal.pone.0091776

Figure S4 Antifungal activity of HRDs against *C. albicans* and *F. graminearum*

(A) *C. albicans* and (B) *F. graminearum* were grown in the presence of HRDs as well as the well characterised defensin NaD1. Growth was assessed by reading the absorbance at 595 nm and presented relative to the untreated control. Data is representative of three independent experiments. Error bars are standard deviation of three technical replicates.

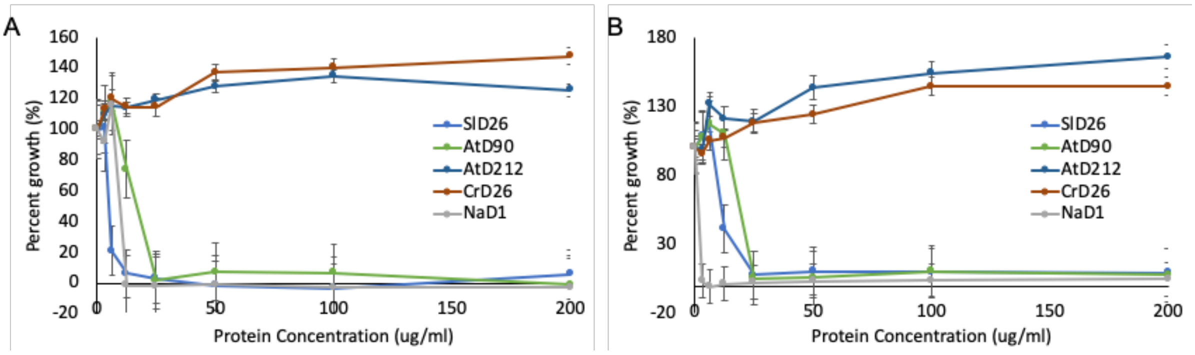

Figure S5: Overlay of the 20 lowest-energy state structures for **A)** SlD26 and **B)** AtD90 with disulphides in yellow, histidines in blue (with their nitrogen atoms in dark blue).

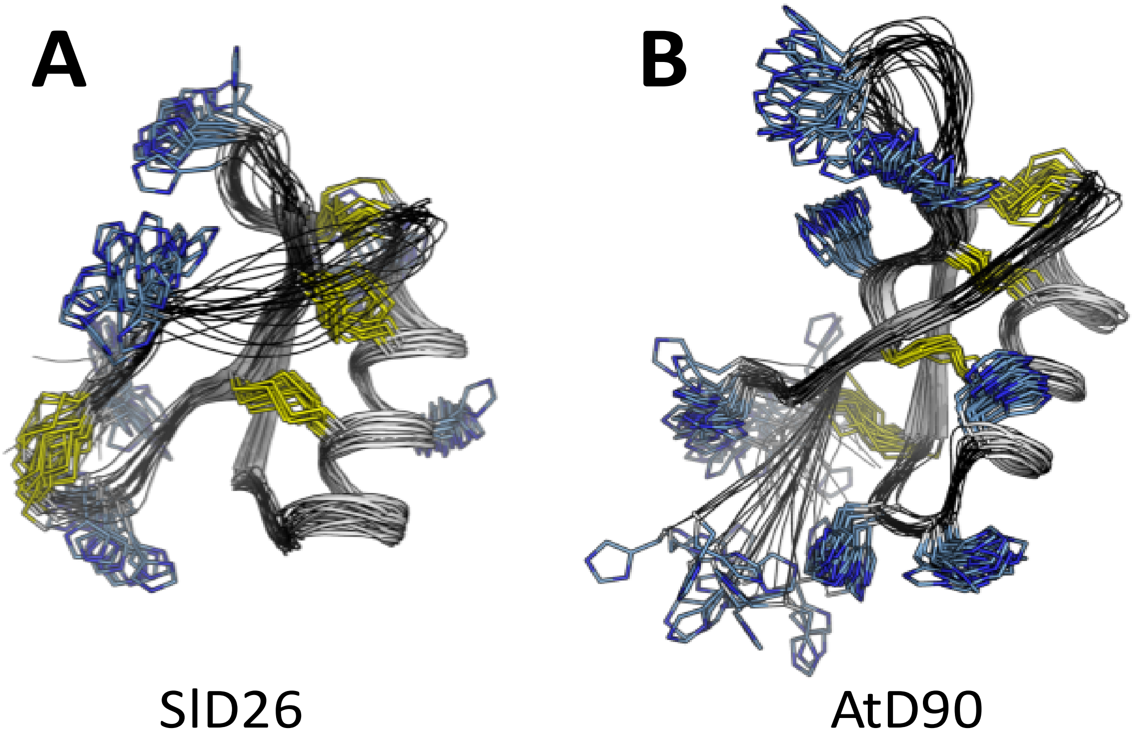

Supplement: Supplementary file 1 [file jof-06-00145-s001.pdf]
